# Bridging the Artificial Intelligence (AI) Gap: A Knowledge, Attitude, and Practice (KAP) Study to Advance Medical Education in Rural Andhra Pradesh

**DOI:** 10.7759/cureus.91822

**Published:** 2025-09-08

**Authors:** Madhulatha Gopidesi, R Anil, Singiri Mahesh, Chitra Nagaraj, Janakiraman Pichandi

**Affiliations:** 1 Community Medicine, People’s Education Society Institute of Medical Sciences and Research, Kuppam, IND; 2 Preventive Medicine, People’s Education Society Institute of Medical Sciences and Research, Kuppam, IND; 3 Biostatistics, People’s Education Society Institute of Medical Sciences and Research, Kuppam, IND

**Keywords:** artifical intelligence, artificial intelligence and education, education and training of medical students and doctors (specialist and phd)), knowledge attitude and practice, medical education curriculum

## Abstract

Background: artificial intelligence (AI) refers to the use of computers and technology to simulate intelligent behavior and critical thinking comparable to humans. During the course of medicine, AI aids medical students in various tasks like studying theory, practical applications, seminar presentations, proposal creation, etc. Understanding and adapting to these advancements is crucial for medical students to enhance their medical knowledge and also to enhance time efficiency.

Objectives: This study aimed to assess the knowledge, attitude, and practice (KAP) regarding AI tools among medical students in rural Andhra Pradesh.

Materials and methods: A cross-sectional study was conducted over two months (June and July 2024) at a rural medical college in Southern Andhra Pradesh. Using systematic random sampling, 210 medical students were selected as participants. After obtaining informed consent, data were collected through an online survey and analyzed using IBM Corp. Released 2014. IBM SPSS Statistics for Windows, Version 23.0. Armonk, NY: IBM Corp.

Results: The study revealed that 84.5% (169/210) of study subjects were using at least one AI tool in medical studies. About 61.4% of study subjects had good knowledge about AI tools being used for studying medicine. It was found that 93.8% of the study subjects had a positive attitude towards the use of AI tools during the course of medicine. It was noted that 89.5% of them were using only four AI tools for medical studies, with the most common being used for the preparation of PowerPoint presentations (34%). The most commonly used AI tools were ChatGPT (OpenAI, California, USA), Meta AI (California, USA), Snap AI (California, USA), and Google Gemini (California, USA), in descending order.

Conclusion: The students have good knowledge and favorable attitudes towards AI tools, but their practical application was narrow in scope, with most students using only a limited set of tools for specific academic purposes. Improving knowledge may enhance practical use among them. A module on the use of AI in medical studies should be integrated into the medical curriculum.

## Introduction

Globally, the adoption of AI in healthcare has increased rapidly, with the market valued at USD 22.4 billion in 2023 and projected to reach USD 188 billion by 2030. AI has been integrated into radiology, diagnostics, documentation, and remote patient monitoring in various forms [1-4]. In India, the AI healthcare market is projected to reach USD 1.6 billion by 2025, with 76% of healthcare professionals being optimistic about its potential to improve patient outcomes [5,6]. Leading institutions in the country are using AI to automate diagnostics and clinical documentation [7], while government initiatives like the Ayushman Bharat Digital Mission integrate AI into health record systems [8]. AI is also being applied in public health and research, from predictive imaging in cardiology [9] to AI-based maternal health outreach programs [10]. Hence, exposure to AI tools is a must for current medical students. Artificial intelligence (AI) is rapidly transforming medical education, offering new opportunities for enhancing learning, research, and clinical decision-making. AI tools such as ChatGPT, Google Gemini, and other large language models can support information retrieval, summarization, and content generation, making them valuable for medical students. However, the surge in AI availability has raised questions about students’ ability to use these tools effectively, ethically, and in alignment with curricular objectives. Several studies [11-13] have been conducted across countries such as Pakistan, Jordan, and Kuwait to understand the knowledge, attitudes, and practices (KAP) of medical students regarding AI tools. Still, there is limited evidence from rural Indian medical colleges. Differences in infrastructure, faculty training, and curriculum integration between urban and rural institutions may influence both access and application. Rural institutions are catching up quickly, but they face unique challenges, including limited internet connectivity, fewer AI-related training opportunities, and a shortage of faculty expertise. Understanding how students in these settings engage with AI is crucial for designing effective curricular interventions and preventing a digital skills gap within the medical workforce.

Therefore, this study was conducted with an objective: to assess the knowledge, attitude, and practice (KAP) regarding artificial intelligence (AI) tools among MBBS (Bachelor of Medicine, Bachelor of Surgery) students in a rural medical college in Andhra Pradesh, India. By identifying current levels of awareness and use, the findings aim to guide targeted educational interventions and inform curriculum development.

## Materials and methods

Study design

This is a cross-sectional study.

Study setting

The study was conducted at PES Institute of Medical Sciences & Research (PESIMSR) located in Kuppam, Andhra Pradesh.

Study period

The study was conducted during June and July 2024.

Study population

The study population consisted of MBBS students from PESIMSR, Kuppam.

In India, the MBBS (Bachelor of Medicine, Bachelor of Surgery) program is the primary undergraduate medical degree, equivalent to the MD program in countries such as the United States. It is typically a 5.5-year course, including a one-year compulsory rotating internship. The second, third, and fourth years of MBBS (the second year represents phase 2, the third year represents phase 3: part 1, and the fourth year represents phase 3: part 2) correspond to clinical training phases where students are exposed to both theoretical and practical aspects of medicine, similar to the preclinical and clinical years of MD programs internationally.

Sampling method

Systematic random sampling method: The sampling frame consisted of all 450 MBBS students enrolled in the second, third, and fourth years at PESIMSR during the study period. A list of these students was obtained from the academic office and served as the sampling frame. Using systematic random sampling, every kᵗʰ student was selected, where k was determined by dividing the total number of eligible students (450) by the required sample size (210). The kᵗʰ number was two. The first student was chosen by the lottery method, and subsequent participants were selected at fixed intervals from the ordered list until the target sample size was reached.

Sample size

Assuming the prevalence of knowledge on AI among medical students is 50% and applying the Z-value for a 95% CI of 1.96 and a precision of 7%, the sample size was calculated as 196. However, a total of 210 students were included in the study.

Inclusion criteria

The study included medical students in the second, third, and fourth years of the MBBS course.

Exclusion criteria

Medical students who did not give consent were excluded from the study.

Study tool

A structured, semi-validated proforma questionnaire was developed using Google Forms. The questionnaire was designed to assess the knowledge, attitude, and practice (KAP) regarding artificial intelligence (AI) tools among medical students. The initial draft of the questionnaire was prepared based on a review of relevant literature, including previously validated KAP studies on AI in medical education [11]. Certain items were adapted or modified to suit the local context and the target population. Content and face validity were ensured through expert review by three faculty members from the Department of Community Medicine. The final questionnaire included:

Knowledge

Five multiple-choice questions were administered, with scores categorized as poor (0-2), moderate (3-4), and good (5).

Attitude

Five multiple-choice questions were scored and categorized as poor (≤12), moderate (13-19), and good (≥20).

Practice

Eleven questions were scored and categorized as poor (≤12), moderate (13-18), and good (≥19).

The complete questionnaire can be accessed through the Appendix to this article.

Method of data collection

The student participants were met in the classroom, and the purpose of the study was explained to them. An online form was sent to each participant, which they then filled out and submitted at their leisure. The online forms also contained the consent form.

Statistical analysis of data

The data were entered in MS Excel 2016 and further analyzed using IBM Corp. Released 2014. IBM SPSS Statistics for Windows, Version 23.0. Armonk, NY: IBM Corp. Descriptive statistics were used. Categorical data were analyzed using frequency and percentages, and the continuous data were analyzed using mean and standard deviation. The association between demographic factors and knowledge, attitude, and practice scores was found using the chi-square test. There were no missing data, as all the details collected through online forms were mandatory for the participants who consented to participate in the study.

## Results

Among the 210 medical students who participated in the study, the mean age was 21.5 ± 1.2 years, with a range of 19 to 26 years. The sample consisted of 122 females (122 [58.1%]) and 88 males (88 [41.9%]). Students were evenly distributed across the second, third, and fourth years of the MBBS program, with 70 students (33.3%) in each academic phase. A large majority (169 [84.5%]) reported having used at least one artificial intelligence (AI) tool in their academic studies.

Knowledge of AI tools

**Figure 1:**
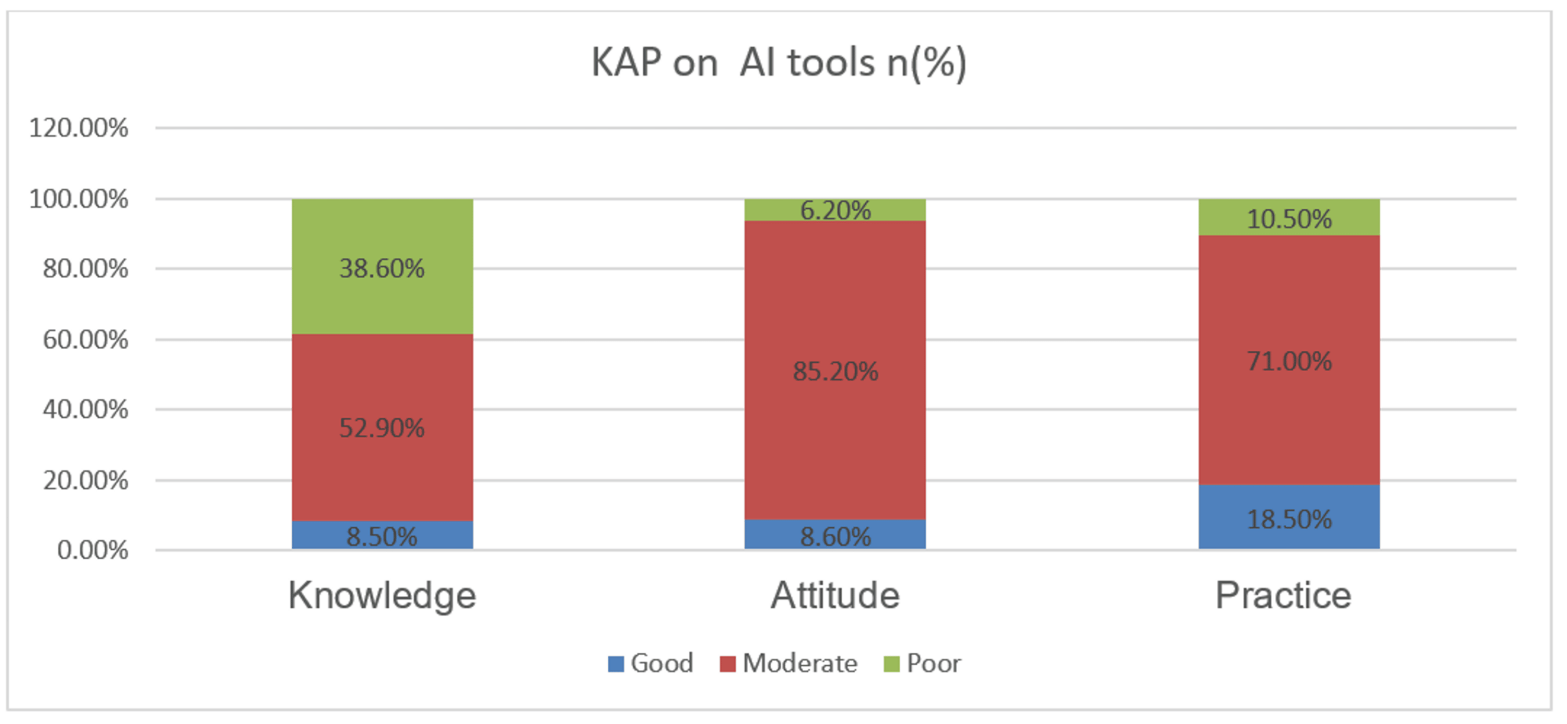
Distribution of study population based on knowledge, attitude, and practices of AI tools (N =210)

As illustrated in Figure 1, 129 participants (129 [61.4%]) demonstrated moderate to good knowledge of AI tools, while 81 students (81 [38.6%]) had poor knowledge. The association between knowledge level and demographic variables is presented in Table 1. Slightly better knowledge was observed among students older than 20 years (16 [9.3 %]) and those in Phase IV (10 [4.3 %]). However, none of the associations with gender, age group, or academic phase were statistically significant (p > 0.05), suggesting that knowledge levels were fairly uniform across the study population.

**Table 1:** Association of demographic factors with knowledge in AI tools A *p-value of <0.05 will be considered as statistically significant.

Knowledge		Poor n (%)	Moderate n (%)	Good n (%)	χ² value	P-value
Gender	Female	51 (41.80%)	59(48.36%)	12 (9.84%)	2.4452	0.294
	Male	30 (34.09%)	52 (59.09%)	6(6.82%)		
Age	<20years	21 (55.26%)	15 (39.47%)	2 (5.26%)	5.5159	0.063
	>20years	60 (34.88%)	96 (55.81%)	16 (9.30%)		
Phase	Phase II	31 (44.29%)	33 (47.14%)	6 (8.57%)	8.0561	0.090
	Phase III	29 (41.43%)	39(55.71%)	2 (2.86%)		
	Phase IV	21(38.57%)	39 (55.71%)	10 (14.29%)		

Attitude towards AI tools

A substantial majority of the students (197 [93.8%]) expressed a positive (moderate to good) attitude toward the integration of AI tools in medical education, while only 13 students (13 [6.2%]) showed a poor attitude, as shown in Figure 1. The association between attitude and background variables is detailed in Table 2. A statistically significant association was found between gender and attitude (p = 0.021), with more male students (12 [13.6%]) expressing a good attitude compared to female students (6 [4.9%]). This may reflect greater confidence or willingness to engage with emerging technologies among male students. However, age (p = 0.204) and academic phase (p = 0.074) were not significantly associated with attitude levels.

**Table 2:** Association of demographic factors with attitude in AI tools A *p-value of <0.05 will be considered statistically significant.

Attitude		Poor n (%)	Moderate n(%)	Good n (%)	χ² value	p-value
Gender	Female	5(4.10%)	111(90.98%)	6 (4.92%)	7.7195	0.021*
	Male	8 (9.09%)	68 (77.27%)	12(13.64%)		
Age	<20years	0 (0.00%)	34(89.47%)	4 (10.53%)	3.1766	0.204
	>20years	13(7.56%)	145(84.30%)	14 (8.14%)		
Phase	Phase II	7 (10.00%)	54 (77.14%)	9 (12.86%)	8.5256	0.074
	Phase III	5(7.14%)	59(84.29%)	6(8.57%)		
	Phase IV	1(1.43%)	66(94.29%)	3(4.29%)		

Practice and usage of AI tools

Concerning practical application, 188 students (188 [89.5%]) reported regular use of AI tools, while 22 (22 [10.5%]) had never used them, as shown in Figure 1. The association between practice behavior and demographic variables is presented in Appendix. A statistically significant association was found between the academic phase and AI practice (p = 0.044), with only nine final-year students (9 [12.9%]) showing good practice compared to 16 students (16 [22.9%]) in Phase II and 14 (14 [20.0%]) in Phase III. Gender (p = 0.700) and age (p = 0.789) were not significantly related to AI practice patterns.

AI tool preferences

**Figure 2:**
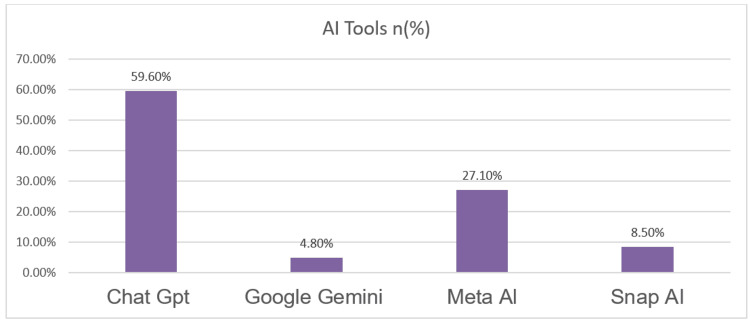
Distribution of study population based on the AI tool frequently used (N =188)

Among the 188 students who reported using AI tools, ChatGPT was the most frequently used, with 112 participants (59.6%) preferring it. Meta AI was used by 51 students (51 [27.1%]), followed by Snap AI (16 [8.5%]) and Google Gemini (9 [4.8%]), as shown in Figure 2. No other AI tools were mentioned.

Purpose of AI tool usage

**Figure 3:**
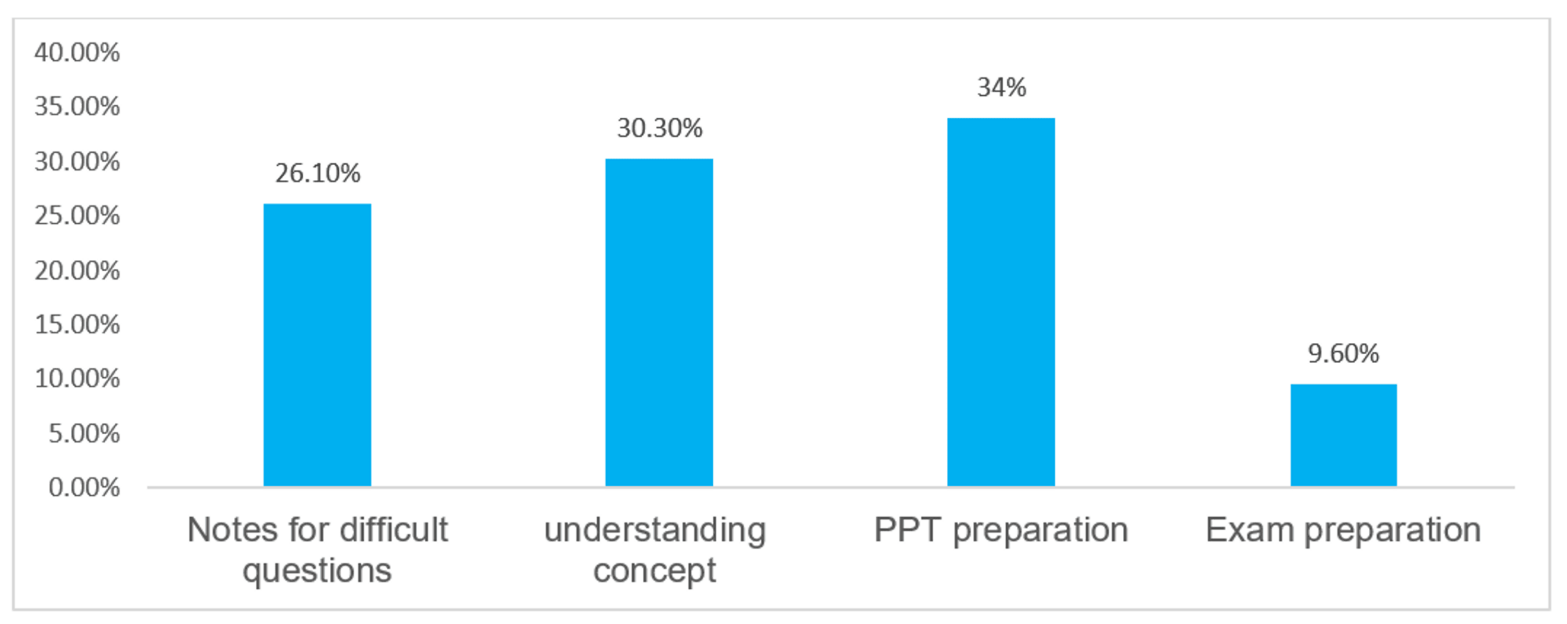
Distribution of study population based on the activity for which the AI tool is used (N =188)

The primary purposes for which students used AI tools are shown in Figure 3. The most common uses were for preparing PowerPoint presentations (64 [34.0 %]), understanding complex concepts (57 [30.3 %]), and note-taking for complex topics (49 [26.1 %]). Exam preparation was the least reported reason (18 [9.6 %]).

## Discussion

Artificial Intelligence (AI) has rapidly transformed various domains, including medical education, by enhancing learning, research, and clinical decision-making. As AI tools become more accessible, it is crucial to understand their adoption, knowledge, attitude, and practice among medical students.

In this study, a majority of students demonstrated moderate to good knowledge of AI, reflecting growing awareness but also exposing significant gaps. Similar findings were reported in Jordan (49.5%) [12] and in a recent systematic review [14], which highlighted moderate exposure across medical curricula worldwide. One likely explanation for this only moderate level of knowledge is the absence of structured AI training within the MBBS curriculum, forcing students to rely on informal sources such as peers and online platforms. The fact that more than one-third of students still had poor knowledge underscores missed opportunities for systematic instruction. These findings suggest that unless AI competencies are explicitly incorporated into the medical curriculum, knowledge will remain fragmented and inadequately translated into practice.

Nearly all participants in this study expressed a positive attitude toward AI tools, consistent with reports from Kuwait and other regions [13]. While this enthusiasm is encouraging, educational theory such as the Technology Acceptance Model (TAM) suggests that favorable attitudes alone do not ensure adoption or effective use. The gap between high attitude scores and more limited practice may be due to barriers such as a lack of institutional guidance, limited faculty encouragement, or uncertainty about how AI can be responsibly applied in learning and clinical contexts. This indicates that while students are conceptually receptive, structured training modules and supervised exposure are needed to transform enthusiasm into meaningful competence.

Although most students reported using AI tools, their scope of practice was narrow, with only four tools (ChatGPT, Meta AI, Snap AI, and Google Gemini) in active use. ChatGPT dominated due to its accessibility and versatility, but applications were largely confined to basic academic tasks like preparing presentations and notes. More advanced uses, such as research assistance, clinical simulations, or decision-making support, were rarely reported. Compared with the Jordan study, which found a higher proportion of non-users [12], these results suggest greater uptake in our setting, but with underutilization of AI’s broader potential. Barriers such as limited awareness of available tools, internet connectivity issues, or even discouragement from faculty may contribute to this restricted adoption.

While students in this study mainly used AI for preparing presentations, clarifying concepts, and note-taking, the potential applications extend much further. To align with these findings and support broader adoption, we recommend integrating AI into the medical curriculum through structured modules. Various modules that demonstrate the application of AI in study planning, including a flashcard generator, model exam question creator, memory aids, etc., can be introduced to the medical students. Integration of AI in medical education will also benefit the students in proposal writing and as a platform for differential diagnosis discussions. Embedding such applications into workshops or elective modules would allow students to progress from surface-level use toward meaningful, competency-based application of AI in both academic and clinical settings.

The analysis of demographic factors, such as gender, age, and phase of study, reveals no statistically significant association with knowledge of AI tools among university students, indicating relatively uniform AI literacy among this population. At the same time, male students may demonstrate slightly higher familiarity: Lamrabet [15] reports these differences are not statistically significant. Similarly, age shows a mild positive correlation with AI knowledge; however, research by Tin [16] and Moravec [17] confirms that this trend lacks statistical significance. The academic phase, whether first-year or final-year, also does not significantly affect AI knowledge levels, as noted by Nikoulina and Caroni [18]. However, some studies suggest that demographic factors may influence students' attitudes toward AI, even if their actual knowledge remains comparable. The relationship between gender and attitudes toward AI in medicine reveals notable differences, with male students generally displaying greater confidence and more favorable views toward adopting the technology [19].

In contrast, female students, though supportive, often express more caution regarding AI's role in healthcare. Studies show that age and academic phase do not significantly affect attitudes, indicating a consistent perspective among students, regardless of their level of study [20]. Increased clinical exposure among senior students appears to enhance their engagement with AI, highlighting academic experience as a key factor in shaping practical use [21]. Although gender plays a role in influencing attitudes, the absence of significant differences in AI practice across demographic lines suggests that structured academic exposure is more influential [22]. These insights underscore the importance of incorporating early, structured AI education into medical training to ensure that all students, regardless of their background, develop confidence and competence in utilizing AI technologies. 

Limitations

This study has certain limitations. First, as a cross-sectional design, it captures students’ knowledge, attitudes, and practices (KAP) at a single point in time, making it difficult to assess trends or establish cause-and-effect relationships. Second, the research was conducted in a single rural medical college in Andhra Pradesh, which may limit the generalizability of the findings to other institutions, particularly urban medical colleges. Third, the study relied on an online self-administered questionnaire, which may have introduced several biases. Non-response bias may have affected representativeness if students who were less interested in AI chose not to participate. Additionally, all measures were self-reported, which may have led to overestimation of actual competence and usage due to social desirability bias. Finally, although the questionnaire was semi-validated and piloted, it contained a limited number of items, which may not reflect the full breadth of AI applications in medical education.

## Conclusions

The findings of this study indicate that medical students in a rural medical college demonstrated moderate to good knowledge of AI tools, a highly favorable attitude, and active but narrow patterns of use. Medical education bodies should develop elective modules or short training courses that cover both theoretical foundations and hands-on applications of AI for MBBS students to translate favorable attitudes into meaningful competence. This exposure will ensure that students not only remain enthusiastic about AI but also acquire the practical skills necessary to apply these tools responsibly in academic and clinical contexts.

## Appendices

**Table 3:** A questionnaire was used to assess knowledge, attitude, and practice with regard to AI tools among medical students. “Credits: Dr. Madhulatha Gopidesi, Dr. Anil R, Dr. Mahesh S, Dr. Chitra Nagaraj" Reference: Ahmed Z, Bhinder KK, Tariq A, Tahir MJ, Mehmood Q, Tabassum MS, Malik M, Aslam S, Asghar MS, Yousaf Z. Knowledge, attitude, and practice of artificial intelligence among doctors and medical students in Pakistan: A cross-sectional online survey. Annals of Medicine and Surgery. 2022 Apr 1;76:103493. https://doi.org/10.1016/j.amsu.2022.103493

Personal details:	Responses
Age	
Gender	
Phase	
Questions Related to Knowledge	
How familiar are you with AI tools in general?	Yes/No
Are you aware of any AI tools specifically designed for medical studies?	Yes/No
Can you name any AI-based tools that assist with studying or learning in medicine? (Open-ended)	
Which of the following is a potential benefit of using AI tools in medical studies?	a) Enhanced Learning and Training b) Increased data silos c) limited access to information d) reduced collaboration opportunities
Can an AI tool generate wrong information?	Yes/No
Questions related to Attitude	
How important do you think AI is in modern medical studies?	A) Not at all important B) Slightly important C) Moderately important D) Very important E) Extremely important
Do you believe AI can improve your learning outcomes?	A) Strongly disagree B) Disagree C) Neutral D) Agree important E) Strongly Agree
Do you think AI will eventually replace traditional study methods?	A) Strongly disagree B) Disagree C) Neutral D) Agree important E) Strongly Agree
How motivated are you to learn more about AI and its applications in medicine?	A) Not at all important B) Slightly important C) Moderately important D) Very important E) Extremely important
I think clinical diagnosis can be made easily using AI tools.	A) Strongly disagree B) Disagree C) Neutral D) Agree important E) Strongly Agree
Questions Related to Practice	
How often do you use AI-based tools for your studies?	A) Never B) Occasionally C) Sometimes D) Often E) Always
How many AI-based tools or applications do you currently use for your studies?	A) None B) 1-2 C) 3-4 D) 5 or more?
How comfortable are you integrating AI tools into your daily routine?	A) Not at all comfortable B) Moderately comfortable C) comfortable D) very comfortable E) Extremely comfortable
Which AI tools or applications do you use most frequently? (Open ended)	
What types of Study tasks do you use AI tools for?	A) Text preparation B) PPT preparation C) Notes for difficult questions D) References
How satisfied are you with the quality of response that you receive from AI tools?	A) Strongly disagree B) Disagree C) Neutral D) Agree important E) Strongly Agree
Do you think AI tools are easy to use?	Yes/No
I have had privacy or security concerns while using AI tools?	A) Strongly disagree B) Disagree C) Neutral D) Agree important E) Strongly Agree
I feel AI tools can reduce my workload.	A) Strongly disagree B) Disagree C) Neutral D) Agree important E) Strongly Agree
Have you experienced any challenges or barriers when using AI tools?	Yes/No
If yes, please specify: (Open-ended)	
https://creativecommons.org/licenses/by/4.0/	
